# A century of intermittent eco‐evolutionary feedbacks resulted in novel trait combinations in invasive Great Lakes alewives (*Alosa pseudoharengus*)

**DOI:** 10.1111/eva.13063

**Published:** 2020-08-25

**Authors:** Shelby E. Smith, Eric P. Palkovacs, Brian C. Weidel, David B. Bunnell, Andrew W. Jones, Devin D. Bloom

**Affiliations:** ^1^ Department of Biological Sciences Western Michigan University Kalamazoo MI USA; ^2^ Department of Ecology & Evolutionary Biology University of California Santa Cruz CA USA; ^3^ United States Geological Survey (USGS) at the Great Lakes Science Center Lake Ontario Biological Station Oswego NY USA; ^4^ United States Geological Survey (USGS) at the Great Lakes Science Center Ann Arbor MI USA; ^5^ National Oceanic and Atmospheric Administration (NOAA) Fisheries Northeast Fisheries Science Center Narragansett RI USA; ^6^ Institute of the Environment and Sustainability Western Michigan University Kalamazoo MI USA

**Keywords:** body shape, eco‐evolutionary dynamics, introduced species, migration, parallel evolution

## Abstract

Species introductions provide opportunities to quantify rates and patterns of evolutionary change in response to novel environments. Alewives (*Alosa pseudoharengus*) are native to the East Coast of North America where they ascend coastal rivers to spawn in lakes and then return to the ocean. Some populations have become landlocked within the last 350 years and diverged phenotypically from their ancestral marine population. More recently, alewives were introduced to the Laurentian Great Lakes (~150 years ago), but these populations have not been compared to East Coast anadromous and landlocked populations. We quantified 95 years of evolution in foraging traits and overall body shape of Great Lakes alewives and compared patterns of phenotypic evolution of Great Lakes alewives to East Coast anadromous and landlocked populations. Our results suggest that gill raker spacing in Great Lakes alewives has evolved in a dynamic pattern that is consistent with responses to strong but intermittent eco‐evolutionary feedbacks with zooplankton size. Following their initial colonization of Lakes Ontario and Michigan, dense alewife populations likely depleted large‐bodied zooplankton, which drove a decrease in alewife gill raker spacing. However, the introduction of large, non‐native zooplankton to the Great Lakes in later decades resulted in an increase in gill raker spacing, and present‐day Great Lakes alewives have gill raker spacing patterns that are similar to the ancestral East Coast anadromous population. Conversely, contemporary Great Lakes alewife populations possess a gape width consistent with East Coast landlocked populations. Body shape showed remarkable parallel evolution with East Coast landlocked populations, likely due to a shared response to the loss of long‐distance movement or migrations. Our results suggest the colonization of a new environment and cessation of migration can result in rapid parallel evolution in some traits, but contingency also plays a role, and a dynamic ecosystem can also yield novel trait combinations.

## 1 INTRODUCTION

The introduction of non‐native species to new environments is a widespread concern (Pimentel, Lach, Zuniga, & Morrison, 2000; Mazzotti, Briggs‐Gonzalez, and Eckles, 2015; Seebens et al., 2017) with considerable economic and ecological implications. Invasive species can compete with and prey upon native species, disrupt food webs and trophic interactions, and introduce diseases (Simberloff & Stiling, 1996; Ricciardi, Steiner, Mack, & Simberloff, 2000; Crowl, Crist, Parmenter, Belovsky, & Lugo, 2008). Despite their many adverse effects, non‐native species also present opportunities for studying rates and patterns of contemporary evolution within their new environments (Willoughby, Harder, Tennessen, Scribner, & Christie, 2018; Gleditsch & Sperry, 2019). Colonizing a new environment may require shifts in species ecology and life history strategy, particularly in migratory species that sever their migratory pathway following the colonization event (Roff, 1991; Palkovacs & Post, 2008; Palkovacs, Dion, Post, & Caccone, 2008; Palkovacs, Mandeville, & Post, 2014; Post, Palkovacs, Schielke, & Dodson, 2008).

Migration is a widespread behavior among animals (Dingle, 1996), and migratory patterns can range from diel vertical migrations in pursuit of food or avoidance of predators to annual migrations between breeding grounds and overwintering habitats, such as the astounding 56,000‐mile trip made by the Arctic tern (*Sterna paradisaea)* (Fijn, Hiemstra, Phillips, & Winden, 2013). The evolution of migration often involves profound phenotypic changes as natural selection optimizes morphological traits for long‐distance movement (Roff, 1988; Bloom, Burns, & Schriever, 2018; Velotta, McCormick, Jones, & Schultz, 2018; Burns & Bloom, 2020). Just as migration can influence the morphology and physiology of an organism, the cessation of migration can, in turn, shift the adaptive optima and drive life history evolution of populations (Morita, Yamamoto, & Hoshino, 2000; Chapman, Brönmark, Nilsson, & Hansson, 2011; Ohms, Sloat, Reeves, Jordan, & Dunham, 2014; Gillanders, Izzo, Doubleday, & Ye, 2015). Adaptive shifts associated with the loss of migration can alter species ecologies, such as changes in trophic niche or habitat occupancy (Palkovacs & Post, 2008; Palkovacs et al., 2008, 2014; Post et al., 2008; Ostberg, Pavlov, & Hauser, 2009; Jones, Palkovacs, & Post, 2013). However, gaining a detailed understanding of the response of a species to new selective pressures in a novel environment (i.e., losing the ability to migrate, such as anadromous migratory species becoming landlocked) is challenging because historical data needed to track changes over time are rarely available.

Natural history collections often play a key role in tracing evolutionary responses to changing or new environments because these institutions catalog specimens over a historical time series. For instance, in a study by Geladi et al. (2019), museum specimens revealed how two fishes native to a Panamanian lake, *Astyanax ruberrimus* and *Roeboides* spp., responded to anthropogenic pressures and the introduction of a non‐native predatory fish species over a 100‐year period. Blanke, Chikaraishi, and Vander Zanden (2018) documented changes in niche breadth and diet shift of deepwater coregonines in the Laurentian Great Lakes over a 100‐year time span. Another study by Kern and Langerhans (2018) analyzed museum specimens over a 50‐year period to highlight rapid morphological adaptation in *Rhinichthys obtusus* and *Semotilus atromaculatus* when exposed to anthropogenically altered stream hydrology. Des Roches et al. (2019) used historical collections to show that climate‐driven habitat change has shaped threespine stickleback (*Gasterosteus aculeatus*) evolution in California estuaries over the past 40 years. In this study, we used museum and contemporary specimens of alewives (*Alosa pseudoharengus*) to investigate how introduced populations of this species adapted to a novel environment in the Laurentian Great Lakes, which are effectively landlocked from the Atlantic Ocean for alewives.

Alewives are native to the Atlantic Coast in North America, with a range extending from the Gulf of St. Lawrence and Nova Scotia to North Carolina (Whitehead, 1985). In their native range, alewives include anadromous populations that migrate from the ocean into freshwater to spawn (Kissil, 1974; Loesch, 1987) and populations that have become landlocked in freshwater lakes from natural damming, anthropogenic damming, and stocking over the past 350 years (Palkovacs et al., 2008; Twining & Post, 2013). Previous studies found that each landlocked population is genetically distinct and the result of independent colonization events, while anadromous populations show population structure across the anadromous range but also high rates of gene flow between neighboring rivers (Palkovacs et al., 2008; Reid et al., 2018). Landlocked alewives in their native range are known to attain sexual maturity at an earlier age and smaller size, have lower fecundity, and grow more slowly (Graham, 1956). Additionally, landlocked alewives spawn at later time and over a longer duration than migratory life history variants (Littrell et al., 2018), although Reid et al. (2019) documented hybridization between the forms following secondary contact. Several studies have investigated phenotypic variation among East Coast anadromous and landlocked populations and found that the landlocked populations exhibit parallel evolution in traits associated with trophic niche and locomotion (Palkovacs & Post, 2008; Palkovacs et al., 2008, 2014; Post et al., 2008; Jones et al., 2013). In each respective landlocked population, alewives rapidly depleted larger‐bodied zooplankton (Brooks & Dodson, 1965; Palkovacs, 2007; Palkovacs & Post, 2008; Post et al., 2008), ultimately restructuring zooplankton communities to predominantly small‐sized zooplankton species. These landlocked populations revealed a classic example of an eco‐evolutionary feedback loop in which size‐selective feeding of the alewives resulted in smaller available zooplankton species, which in turn drove the evolution of smaller gill raker spacing and narrower gape width in alewives (Palkovacs & Post, 2008; Jones et al., 2013; Palkovacs et al., 2014). In contrast, the East Coast anadromous population restructured lake zooplankton communities seasonally, but the outmigration of alewives to the ocean allowed large‐bodied zooplankton communities to rebound, resulting in a stable zooplankton community composition over time, thereby preventing strong feedback on the evolution of alewife foraging traits (Palkovacs & Post, 2008). As a result, the anadromous population maintained larger gill raker spacing and gape width (Palkovacs & Post, 2008; Palkovacs et al., 2008; Post et al., 2008). Independently colonized, landlocked populations showed consistent decreases in body size and parallel body shape evolution (Jones et al., 2013). These repeated parallel patterns suggest a more common generality, namely, that becoming permanently landlocked changes the adaptive landscape and drives rapid phenotypic evolution in response to the loss of a migratory life strategy (Palkovacs & Post, 2008; Palkovacs et al., 2008).

In the Great Lakes, alewives were first documented in Lake Ontario in 1873 (Bean, 1884; Miller, 1957), although the exact date of introduction and pathway is unknown. Hypotheses for the origin of alewives in the Great Lakes include inadvertent stocking with American shad (Emery, 1985; Mills et al., 1993) and passage through the St. Lawrence Seaway (Caspers, 1976) or Erie Canal (Smith, 1970). Some have even speculated that alewives might be native to Lake Ontario but noted that evidence was lacking (Miller, 1957). Despite the uncertainty surrounding their mode of entry into the Great Lakes, alewives likely accessed Lake Erie following the development and enlargement of the Welland Canal and subsequently colonized the remaining Great Lakes (Dymond, 1932; Ihssen, Martin, & Rodgers, 1992; O’Gorman & Stewart, 1999; Lee & Lee, 2017). Alewives were first reported in Lake Erie in 1931 (Dymond, 1932; Ihssen et al., 1992), Lake Huron in 1933 (MacKay, 1934), Lake Michigan in 1949 (Miller, 1957; Brown 1972), and finally Lake Superior in 1954 (Miller, 1957). In several of the Great Lakes, alewife populations grew rapidly (Miller, 1957). For example, alewife densities peaked in Lake Michigan around 1966 (Brown 1972), which was followed by a massive die‐off in 1967 (O’Gorman & Stewart, 1999). Non‐native Coho salmon (*Oncorhynchus kisutch*) and Chinook salmon (*Oncorhynchus tschawytscha*) were also successfully introduced in 1966 and 1967, respectively, in Lake Michigan (Tanner & Tody, 2002) to establish a recreational and commercial sport fishery, which was expected to exploit alewives as a prey resource.

Since the 1960s, a myriad of other aquatic invasive species have also become established in the Great Lakes, with the rate of introduction averaging an astounding one new species every eight months (Ricciardi, 2006). Many of these species, such as filter‐feeding quagga mussels (*Dreissena bugensis*) and zebra mussels (*Dreissena polymorpha*), indirectly compete with alewives by redirecting the flow of primary productivity from the pelagic zone where alewives feed to the littoral‐benthic zones (Hecky et al., 2004). Spiny water flea (*Bythotrephes longiramus*) and fishhook water flea (*Cercopagis pengoi*), conversely, can directly compete with alewives for smaller zooplankton prey but also can serve as prey to larger alewives (Pothoven and Vanderploeg, 2004; Stewart et al., 2009). Therefore, many of the new species introductions potentially altered the evolutionary trajectory of trait evolution in Great Lakes alewives. Moreover, while the East Coast inland lakes range in size from 70 to 422 acres (CT.gov, 2006), Lake Ontario is estimated to be 4.7 million acres, over 10,000 times larger than the largest East Coast inland lake, while Lake Michigan is even larger at an estimated 14.3 million acres (EPA, 2011). Hence, comparing alewife traits among systems that are landlocked but yet offer environmental differences in size and species composition offers a unique research opportunity to understand drivers of trait evolution.

In this study, we analyzed traits associated with foraging and motility, and used geometric morphometrics to quantify changes in body shape. Using these data, we compared phenotypic patterns of evolution between native anadromous and landlocked alewife populations with introduced Great Lakes populations of alewives. Using historical museum and contemporary field‐collected specimens, we characterized phenotypic changes in Great Lakes alewives over the past 95 years. We tested the hypothesis that Great Lakes alewives would exhibit parallel evolution with East Coast landlocked populations in traits associated with the loss of migration (body shape and depth) and that the trophic traits of Great Lakes alewives would mirror those of East Coast landlocked populations and evolve in response to eco‐evolutionary feedbacks present from reshaping freshwater zooplankton communities. Under this hypothesis, we predicted that Great Lakes alewives would similarly evolve smaller gill raker spacing and gape width in response to a decrease in large zooplankton availability, and a deeper body shape as a result of the cessation of long‐distance migration.

## 2 METHODS

### 2.1 Specimen acquisition

We used historical museum and contemporary field‐collected specimens to generate a time series of morphological change over time in Great Lakes populations of alewives. Contemporary specimens are defined as the most recent specimens, collected in the 2010s (date range: 2013–2017). We used FishNet2 [http://www.fishnet2.net] to aggregately search natural history collections for Great Lakes alewife records for the earliest possible collection date. Museum records discovered using FishNet2 were augmented with reports of alewife collections from the Great Lakes reported in the literature (Bean, 1884; Miller, 1957). The earliest records (either museum specimens or literature) do not necessarily indicate the precise time of introduction to each lake, but rather the earliest collection date after alewives were established in each lake, respectively. We selected collections (museum lots) from each decade in which at least three, and up to 916 alewife specimens were available. Only fish equal to or greater than 30 millimeters total length were used due to the difficulty involved in extracting gill arches without damaging the gill rakers and in order to correct for allometric size differences during ontogeny, reduce the potential impacts of plasticity, and remain consistent with data available from East Coast populations (Palkovacs & Post, 2008). Our museum searches recovered specimens ranging from years 1880 to 2013, although the oldest specimens we acquired were from 1922 due to handling restrictions. Initial searches indicated a shortage of appropriately sized fish in Lakes Huron, Erie, and Superior, so we limited our data collection to specimens from Lakes Ontario and Michigan.

Contemporary field sampling in Lakes Ontario and Michigan consisted of United States Geological Survey (USGS) bottom trawling surveys. Lake Ontario sampling occurred during an October of 2017 benthic trawl, which consisted of transects sampled along the Southern shore of Lake Ontario off NY (Weidel, Connerton, & Holden, 2018). Trawl duration was approximately 5 minutes and ranged from depths of eight meters up to 220 meters. Fishes were sampled using a 12 meter by 1.5 meter Yankee trawl net. Lake Michigan sampling occurred with the same net type in September of 2017 offshore of Sturgeon Bay, WI, at depths varying from 46 meters to 110 meters. Specimens were initially frozen, then fixed in formalin, and stored in 70‐80% ethanol. Per decade sample sizes, museum identifiers, and available standard lengths of all fish used can be found in Table S1. Samples sizes of Great Lakes specimens varied between foraging trait and body shape analyses because dissection restrictions limited the number of usable specimens in each lot for gill raker spacing and gape width measurements, while body warping and curvature limited usable specimens in geometric morphometric body shape analyses.

### 2.2 Gill raker spacing and gape width measurements

To capture variation in foraging traits of alewives over time, we quantified gape width and gill raker spacing in 261 collective historical and present‐day Great Lakes alewife specimens (*n* = 142 Lake Ontario; *n* = 119 Lake Michigan, Table 1) using identical methods from Palkovacs and Post (2008). Gape width is important for capturing prey; the opening of the mouth and negative pressure created by the buccal cavity suction the prey inward (Wainwright et al., 2007). Gill raker spacing is known to determine size selection of prey items in filter‐feeding fishes (Wright & O’Brien, 1984; Link & Hoff, 1998; Salman, Al‐Mahdawi, & Heba, 2005). Prior to dissection, standard and total lengths of each fish were taken to the nearest millimeter using a Mitutoyo 500‐196‐30 AOS digital caliper. We quantified gape width by opening the mouth of each specimen to its maximum extent and measuring at the greatest horizontal distance. We repeated gape measurements three times and used the average of the three measurements to account for measurement error.

**Table 1:** Sample size of alewives across each decade used in gill raker spacing and gape width analyses. Great Lakes alewives were comprised of museum and contemporary field‐collected specimens, while East Coast anadromous and landlocked data were collected in 2004 and 2005 and provided by Palkovacs and Post (2008)

Decade	Museum specimens from Lake Ontario	Museum specimens from Lake Michigan
1920s	30	‐
1930s	7	‐
1940s	29	‐
1950s	‐	10
1960s	‐	45
1970s	15	30
1980s	12	14
1990s	‐	‐
2000s	‐	6
2010s	49	14
**Great Lakes totals**	**142**	**119**
**Combined Great Lakes total**		**261**

**Life history form**	**Population**	**Individuals**
East Coast anadromous	Bride Lake	56
East Coast anadromous	Dodge Pond	49
East Coast anadromous	Gorton Pond	59
**East Coast anadromous total**		**164**
East Coast landlocked	Crystal Lake	26
East Coast landlocked	Amos Lake	20
East Coast landlocked	Uncas Pond	22
East Coast landlocked	Saltonstall Lake	25
East Coast landlocked	Long Pond	16
East Coast landlocked	Mashapaug Lake	26
East Coast landlocked	Pattagansett Lake	76
East Coast landlocked	Quonnipaug Lake	90
East Coast landlocked	Rogers Lake	80
**East Coast landlocked total**		**381**
**Combined East Coast total**		**545**
**Combined Great Lakes/East Coast total**		**806**

Note

Bold values comprise total samples sizes of combined lakes or populations.

We measured gill raker spacing by first removing the anteriormost branchial arch from the left side of each fish. The anteriormost gill arch is the most well‐developed arch that carries out most of the filtering (MacNeill & Brandt, 1990) and it possesses the longest gill rakers. We photographed dissected gill arches using a Nikon SMZ1500 dissecting microscope equipped with an Infinity Lumen*era* 3 microscope‐mounted camera at 0.75–10× magnification. Gill arches that were too large for the entire arch to fit within the microscope‐mounted camera frame were measured manually using a digital caliper to the nearest 1/100 millimeter. We digitally measured attributes of each gill arch using Infinity Analyze version 6.5 software. We computed gill raker spacing (GRS) according to Palkovacs and Post (2008), which is as follows: GRS = (L– N * W)/N, where N is the overall number of gill rakers, L is the combined lengths of the upper and lower gill arches, and W is the averaged widths of the first gill rakers on the upper and lower gill arches.

We size‐standardized gill raker spacing and gape width to the mean total body length using the equation GRS_t_ = GRS_o_ (TL_t_/TL_o_)^b^, where GRS_t_ represents the size corrected trait value, GRS_o_ is the nontransformed observed trait value, TL_t_ is the target body length represented by the mean overall length in the entire dataset, and TL_o_ is the untransformed observed total body length. We log_10_‐transformed gill raker spacing, gape width, and total body length, and a linear regression was performed for each lake independently to generate allometric scaling constant b from each regression slope. t tests, ANOVA with post hoc Tukey’s HSD, and ANCOVA tests were used on mean‐standardized trait values to analyze differences among decades within the historical Great Lakes populations as well as among contemporary alewife populations in the Great Lakes and in East Coast anadromous and landlocked populations. All statistical analyses were implemented using R version 3.6.1 and RStudio version 1.2.1335. We directly compared measurements of gill raker spacing and gape width in historical and present‐day Great Lakes alewives to measurements from East Coast anadromous and landlocked alewife populations collected in 2004 and 2005 provided by Palkovacs and Post (2008) (*n* = 164 anadromous; *n* = 381 landlocked). Both East Coast anadromous and landlocked alewives were represented by several populations or sampling sites that were determined not to differ significantly, and thus were pooled together. Additionally, although several lakes were sampled for anadromous alewives, they were previously shown to represent a single population (Palkovacs et al., 2008; Reid et al., 2018). Specific localities for all specimens are provided in Table 1.

### 2.3 Geometric morphometric analysis

**Figure 1:**
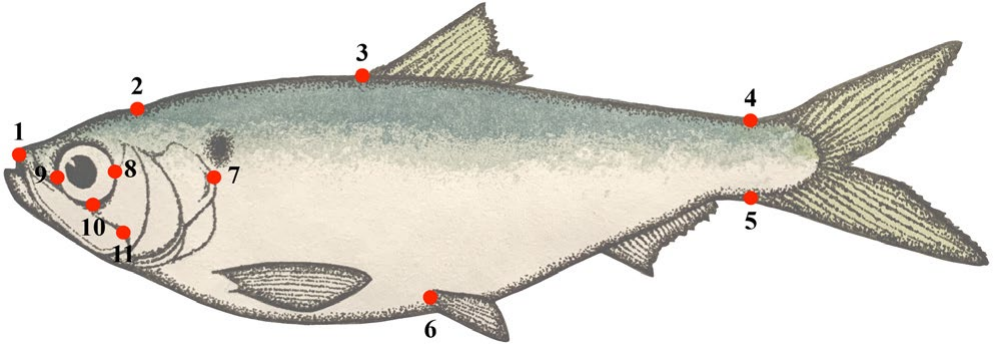
Placement of 11 landmarks to estimate body shape changes using geometric morphometric analyses

We used geometric morphometrics (Bookstein, 1992) to quantify body shape evolution over time in Lake Michigan and Lake Ontario populations, and to compare body shape among four populations: East Coast anadromous, and three landlocked populations: East Coast, Lake Michigan, and Lake Ontario (Table 2). For the latter analysis, we pooled fish from all decades for the Lake Michigan and Ontario populations and used fish collected in 2009 and provided by Jones et al., (2013) for the East Coast anadromous and landlocked populations. Localities of all specimens are provided in Table 2. We photographed each fish on its left side using a Nikon D750 DSLR and used pins and clay to remove all natural concavity from specimens. A metric ruler was included in each shot to allow for allometric standardization. We chose 11 landmarks following Silva (2003) and Jones, Palkovacs, and Post (2013) that are commonly used to capture overall body shape variation in clupeids (Figure 1). Landmarks were placed at (1) the anterior tip of the maxilla, (2) the posterior end of the supraoccipital, (3) the anterior insertion of the dorsal fin, (4) the dorsal insertion of the caudal fin, (5) the ventral insertion of the caudal fin, (6) the anterior insertion of the pelvic fin, (7) the posterior insertion of the operculum, (8) the posterior extent of the orbit, (9) the anterior extent of the orbit, (10) the ventral extent of the orbit, and (11) the posterior extent of the maxilla (Silva, 2003; Jones et al., 2013) using tpsDig2, Release 2.31 (Rohlf, 2010). We selected 377 collective historical and present‐day unwarped Great Lakes individuals (*n* = 176 Lake Ontario; *n* = 201 Lake Michigan) and used 276 photographs of East Coast specimens (*n* = 182 anadromous; *n* = 94 landlocked) from Jones et al. (2013). We reprocessed the photographs of East Coast specimens to mitigate any bias in placement of landmarks as we compared populations. We employed the Procrustes fit function in MorphoJ (Klingenberg, 2011) to generate a consensus shape and prevent variation that can be caused by rotation, translation, and scaling (Rohlf & Slice, 1990). To test for disparity in motility‐associated traits and general body shape between Great Lakes alewives, East Coast anadromous alewives, and East Coast landlocked alewives, we generated a principal component analysis (PCA) on the covariance matrix in MorphoJ. For each ordination, the first two principal components (PCs) summarized at least 52% of the variation in Figure 4, 66 % of the variation in Figure 5, and 51% of the variation in Figure 6. We implemented ANOVA on Procrustes coordinates (shape coordinates) using the function *procD*.*lm* from the R package *geomorph* (Adams & Otárola‐Castillo, 2013) to detect population‐level shape differences. Statistical significance was assessed utilizing 1,000 iterations of a residual randomization permutation procedure.

**Table 2:** Sample size of alewives across each decade used in geometric morphometric body shape analyses. Great Lakes alewives were comprised of museum and contemporary field‐collected specimens, while East Coast anadromous and landlocked data were collected in 2009 and provided by Jones et al. (2013)

Decade	Museum specimens from Lake Ontario	Museum specimens from Lake Michigan
1920s	53	‐
1930s	3	‐
1940s	39	‐
1950s	‐	16
1960s	‐	105
1970s	16	38
1980s	12	21
1990s	‐	‐
2000s	‐	9
2010s	53	12
**Great Lakes totals**	**176**	**201**
**Combined Great Lakes total**		**377**

**Life history form**	**Population**	**Individuals**
East Coast anadromous	Bride Lake	62
East Coast anadromous	Dodge Pond	80
East Coast anadromous	Upper Mill Pond	40
**East Coast anadromous total**		**182**
East Coast landlocked	Pattagansett Lake	44
East Coast landlocked	Quonnipaug Lake	22
East Coast landlocked	Rogers Lake	28
**East Coast landlocked total**		**94**
**Combined East Coast total**		**276**
**Combined Great Lakes/East Coast total**		**653**

Note

Bold values comprise total samples sizes of combined lakes or populations.

## 3 RESULTS

### 3.1 Gill raker spacing

**Figure 2:**
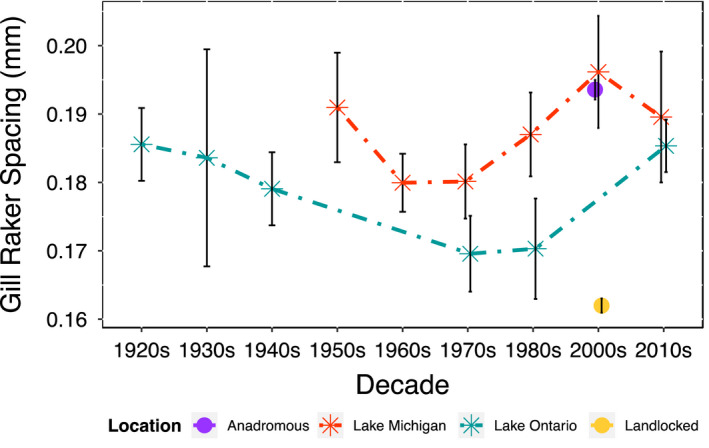
Changes in gill raker spacing (in millimeters) over time in Great Lakes alewife populations and data from the 2000s for East Coast landlocked and anadromous populations. Specimens spanning each decade were combined into a single temporal unit (decade). Sample sizes: *n* = 142 Lake Ontario; *n* = 119 Lake Michigan; *n* = 164 East Coast anadromous; *n* = 381 East Coast landlocked

Significant changes were detected in both Lake Michigan (*p* = .032) and Lake Ontario (*p* = .044) alewife gill raker spacing trajectories over time. Overall, the patterns of Great Lakes alewife gill raker spacing varied over time, with the earliest measurements being similar to anadromous populations, declining until the 1960s in Lake Michigan and 1970s in Lake Ontario, and then increasing to gill raker spacing similar to what was measured in the earliest decades (*p* = .966 and *p* = .916 for Lake Ontario and Lake Michigan, respectively, see Figure 2). Specifically, gill raker spacing for Lake Michigan alewives declined 0.012 millimeters from the 1950s up to the 1960s, while spacing for Lake Ontario alewives declined 0.015 millimeters from the 1920s up until the 1970s. The trajectory for Lake Michigan stabilized between the 1960s and 1970s, while the trajectory for Lake Ontario stabilized a decade later between the 1970s and 1980s. In Lake Michigan, gill raker spacing increased from the 1970s to 2000s and then decreased between the 2000s and 2010s. In Lake Ontario, gill raker spacing increased between the 1980s and 2010s, but we do not have data for the 2000s. When comparing gill raker spacing among the four populations in contemporary times, differences were detected (ANOVA: *F*_3, 604_ = 96.56, *p* < .001), particularly between contemporary Great Lakes populations and East Coast landlocked populations (ANOVA: *F*_2, 441_ = 33.84, *p* < .001). There was no difference detected among contemporary Great Lakes and East Coast anadromous alewives (ANOVA: *F*_2, 224_ = 2.74, *p* = .067), or between present‐day Lake Ontario and Lake Michigan populations (*p* = .633) in gill raker spacing.

### 3.2 Gape width

**Figure 3:**
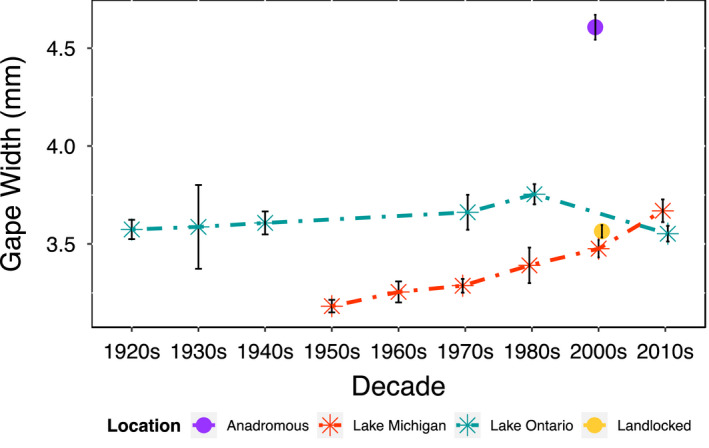
Changes in gape width (in millimeters) over time in Great Lakes alewife populations and data from the 2000s for East Coast landlocked and anadromous populations. Specimens spanning each decade were combined into a single temporal unit (decade). Sample sizes: *n* = 142 Lake Ontario; *n* = 119 Lake Michigan; *n* = 164 East Coast anadromous; *n* = 381 East Coast landlocked

Lake Ontario alewives had a gape width that was similar to East Coast landlocked populations over time, while early Lake Michigan alewives possessed a significantly smaller gape width than East Coast landlocked populations (*p* < .001) and experienced a consistent increase in gape width, eventually matching the gape width of East Coast landlocked alewives (Figure 3). We found significant differences among contemporary Great Lakes populations and East Coast anadromous alewives (ANOVA: *F*_2, 224_ = 48.22, *p* < .001) in gape width. There was no significant difference among contemporary Great Lake populations and East Coast landlocked populations (ANOVA: *F*_2, 439_ = 0.24, *p* = .790), or between contemporary Lake Ontario and Lake Michigan populations (*p* = .152) in gape width. Independently, gape width in historical Lake Ontario alewives remained relatively unchanged across all decades (*p* = .166), while Lake Michigan fish exhibited a significant 0.5‐millimeter gape width increase in each decade from the 1950s to 2010s (*p* = .003). When comparing gape width between the date of initial colonization in each Great Lake and present‐day gape width, only Lake Michigan fish exhibited a significant difference (*p* < .001).

### 3.3 Geometric morphometric analysis

**Figure 4:**
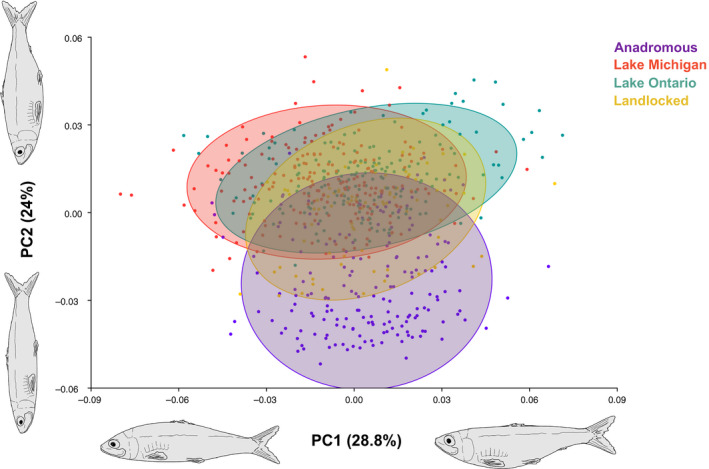
Principal component analysis of body shape data for Great Lakes, East Coast landlocked, and East Coast anadromous alewives. Great Lakes populations include all decades sampled, while East Coast populations are represented by specimens from the 2000s. Alewife illustrations were substituted in place of wireframe grids to depict body change along each principal component

Our principal component analysis showed strong overlap in body shape between Great Lakes populations and East Coast landlocked populations overall, while East Coast anadromous populations differed from both Great Lakes populations and East Coast landlocked populations most significantly along PC2 (Figure 4). PC1 and PC2 characterized 52% of the variation observed among lateral body shape and trait change, with PC1 describing 28.8% of that variation and PC2 describing the remaining 24% of variation. PC1 corresponded with differences in mouth orientation and curvature of the body. East Coast anadromous fish and East Coast landlocked fish possessed a more terminal oriented mouth and intermediate body curvature, while the Lake Michigan fish displayed a more sub‐terminal oriented mouth and dorsally concentrated curvature. Lake Ontario fish displayed a more super‐terminal oriented mouth and ventrally emphasized curvature.

PC2 corresponded with differences in head size, caudal peduncle size, and body depth. The East Coast landlocked fish occupied body shape space between anadromous and Great Lakes landlocked populations, but were more similar to Lake Michigan alewives and Lake Ontario alewives than the East Coast anadromous population for PC2. Individually, the East Coast anadromous population had the most negative PC2 values and displayed a larger head, shallower, more cylindrical body shape, and shorter, thicker caudal peduncle. The East Coast landlocked population was median‐positive situated in morphospace, displaying a smaller head, deeper body, and longer caudal peduncle. The Great Lakes landlocked populations had the most positive PC2 values and displayed a smaller head, deeper, more robust body, and longer, thinner caudal peduncle.

**Figure 5:**
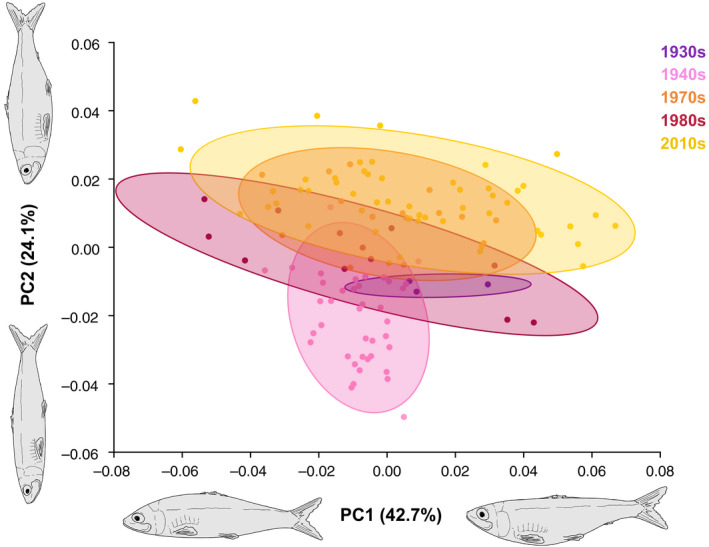
Principal component analysis of body shape data for Lake Ontario alewives. Each respective year in the legend represents specimens from an entire decade. Alewife illustrations along each x‐ and y‐axis indicate body shape changes

**Figure 6:**
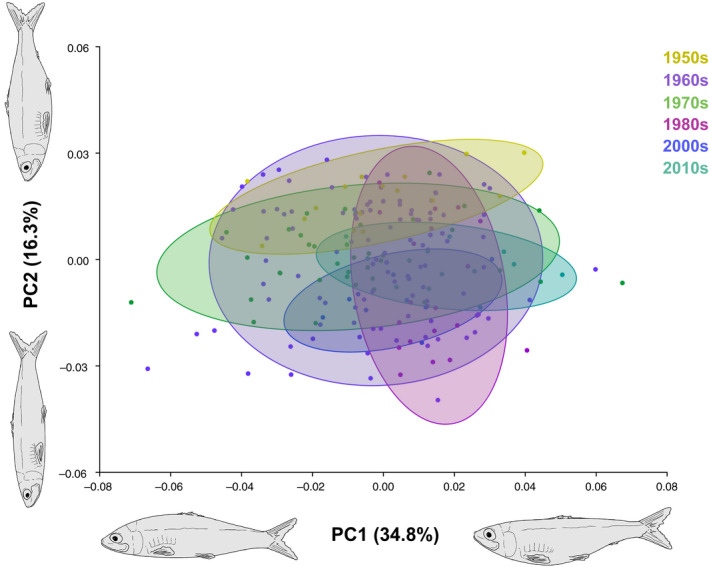
Principal component analysis of body shape data for Lake Michigan alewives. Each respective year in the legend represents specimens from an entire decade. Alewife illustrations along each x‐ and y‐axis indicate body shape changes

We did not detect a clear evolutionary trajectory in body shape changes over a period of 62 years in Lake Michigan alewives (Figure 6), but did find a consistent increase along PC2 in Lake Ontario alewives over 85 years (Figure 5), which describes head size, body depth, and caudal peduncle morphology. Lake Ontario fish shifted from larger heads with shallower bodies and shorter, thicker caudal peduncles in the 1930s and 1940s, to possessing smaller heads, more robust and deeper bodies, and thinner, longer caudal peduncles in the 1970s and 2010s.

Our ANOVA of the Procrustes coordinates revealed significant differences among all four alewife populations (ANOVA: *F*_3, 648_ = 57.79, *p* < .001, Figure 4), including between East Coast anadromous and landlocked populations (ANOVA: *F*_1, 274_ = 55.44, *p* < .001) and between East Coast anadromous and Great Lakes alewife populations (ANOVA: *F*_2, 557_ = 69.91, *p* < .001). Although there was strong overlap among Great Lakes populations and East Coast landlocked alewives along PC1 and PC2 in Figure 4, significant differences were detected between Great Lakes and East Coast landlocked populations (ANOVA: *F*_2, 467_ = 29.94, *p* < .001) and between Lake Ontario and Lake Michigan alewives (ANOVA: *F*_1, 374_ = 21.30, *p* < .001). Additionally, the analyses detected intra‐lake differences across five decades in Lake Ontario (ANOVA: *F*_4, 118_ = 24.59, *p* < .001) and six decades in Lake Michigan (ANOVA: *F*_5, 195_ = 6.15, *p* < .001) (Figure 5 and Figure 6, respectively).

## 4 DISCUSSION

Our study addressed a potential outcome of what happens when migratory fish populations face, and potentially shape, a new adaptive landscape by colonizing a novel environment and becoming permanently landlocked. We showed that alewife colonization of a complex and variable environment in the Great Lakes (Escobar et al. 2018) resulted in novel and dynamic trait combinations. Present‐day gill raker spacing patterns in Great Lakes alewives are consistent with East Coast anadromous populations, while gape width is remarkably similar to East Coast landlocked populations. Although overall body shape in each Great Lakes population differed significantly, alewives in both Great Lakes were more similar to East Coast landlocked populations than East Coast anadromous alewives. This suite of trait combinations is best explained by intermittent eco‐evolutionary feedback loops, which result in shifting adaptive optima over time. Our results demonstrate that traits with different functions show varied responses to the introduction to novel habitats, with a high degree of parallelism in traits related to loss of migration, but more complex responses observed in traits that respond to eco‐evolutionary feedbacks. This result shows that contingency (the role unpredictable events have in shaping future adaptive changes, making them less repeatable; (Losos, Jackman, Larson, De Queiroz, & Rodríguez‐Schettino, 1998; Blount, Lenski, & Losos, 2018)) plays an important role in shaping eco‐evolutionary dynamics in novel environments.

### 4.1 Functional trait evolution and eco‐evolutionary dynamics

Great Lakes alewife gill raker spacing has evolved in response to, but also at times drove, a rapidly shifting plankton community in the Great Lakes over the past 95 years. Alewives are known to preferentially feed on larger zooplankton (Brooks & Dodson, 1965; Palkovacs, 2007; Palkovacs & Post, 2008; Post et al., 2008), and following their colonization in Lakes Ontario and Lake Michigan, the earliest zooplankton tows confirmed that Great Lakes alewives depleted large‐bodied zooplankton stocks, resulting in communities dominated primarily by smaller‐bodied zooplankton (Brown, 1972; Johannsson, 2003; Wells, 1970). For example, in Lake Michigan, zooplankton communities shifted from predominately large cladocerans (e.g., *Daphnia galeata* and *Daphnia retrocurva*), calanoid copepods (e.g., *Epischura lacustris* and *Diaptomus sicilis*), and cyclopoid copepods (e.g., *Mesocyclops edax*) in 1954 to small and medium‐sized zooplankton (e.g., *Daphnia longiremis*, *Bosmina longirostris*, and *Bosmina coregoni*) by 1966 (L. R. Wells, 1970). Lake Ontario experienced an even greater shift from larger to smaller zooplankton assemblages up until the 1970s (Smith 1995). The timing of these shifts from large to small zooplankton size corresponds to a decrease in alewife gill raker spacing from the time of their introduction up until the 1960s (Lake Michigan) and 1970s (Lake Ontario; Figure 2). We suggest that Great Lakes alewives altered zooplankton community structure, which subsequently resulted in a decrease in alewife gill raker spacing as alewives adapted to smaller prey base. This scenario suggests that Great Lakes alewives entered an eco‐evolutionary feedback loop (Palkovacs & Post, 2008; Post et al., 2008; Palkovacs et al., 2014) following initial colonization until the 1970s, a dynamic that parallels the scenario that played out in East Coast landlocked alewives following the construction of colonial era dams and natural landlocking (Palkovacs & Post, 2008; Post et al., 2008; Palkovacs et al., 2014).

After the 1970s in Lake Michigan and 1980s in Lake Ontario, we documented a positive shift in gill raker spacing trajectory in Great Lakes alewives that can likely be traced to several key events. First, as illustrated in Lake Michigan, alewife densities declined after their peak in 1966 due to a massive die‐off (70% of the population, Wells and McLain, 1973). The successful introduction of Coho and Chinook salmon by fishery managers in 1966 and 1967 (Tanner & Tody, 2002) led to further long‐term declines in alewife biomass (Madenjian et al., 2005). We hypothesize that lower alewife densities in the 1970s reduced their ability to structure zooplankton communities and contributed to the recovery of larger‐bodied zooplankters (L. R. Wells, 1970; Crowder, McDonald, & Rice, 1987). The unintentional introduction of dreissenid mussels and large predatory zooplankton were other key events that likely affected the composition of zooplankton prey available to alewife. Invasive spiny water flea were first detected in the Great Lakes in 1982 and reduced the densities of small cladoceran zooplankton (Barbiero & Tuchman, 2004; Pangle, Peacor, & Johannsson, 2007) while also serving as a large prey item for alewives. Likewise, the fishhook water flea was first documented in the Great Lakes in 1998 and filled a similar functional role as the spiny water flea (Mills et al., 1992; Pothoven & Vanderploeg, 2004; Stewart et al., 2009). In fact, several studies have described how the combined effects of introduced dreissenid mussels and predatory cladoceran species affected not only the Great Lakes ecosystem, but also the diets of alewives (Mills et al., 1992; MacIsaac, Lonnee, & Leach, 1995; Pothoven & Madenjian, 2008; Stewart et al., 2009; Vanderploeg et al., 2012; Weidel et al., 2018). For example, Stewart, Sprules, and O’Gorman (2009) described how alewives in Lake Ontario shifted from a diet previously dominated by *Diporeia*, *Daphnia*, and other small zooplankton species in 1972‐1988 to one that relied upon larger zooplankters such as *Mysis* and the introduced predatory cladocerans *B*.*longiramus* and *C*.*pengoi* into the mid‐2000s. With respect to the differences in the timing of the increased spacing between Lakes Michigan and Ontario, we speculate that Lake Ontario alewife populations displayed a later shift due to stocking numbers of Chinook salmon peaking more than a decade after Lake Michigan in the mid‐ to late 1980s (Mills et al., 2003). Regardless, we hypothesize that in both lakes, the reversal in zooplankton size caused the alewives to adapt to favor larger gill raker spacing adapted to capture larger prey. The decrease in alewife abundance and increase in large prey availability likely disrupted the feedback loop that was present pre‐1970s, and explains the increase in gill raker spacing from the 1970s to 2010s. This suggests the complex history of differences between Great Lakes and East Coast landlocked populations is explained in part by the dynamic Great Lakes ecosystem over the past century.

**Figure 7:**
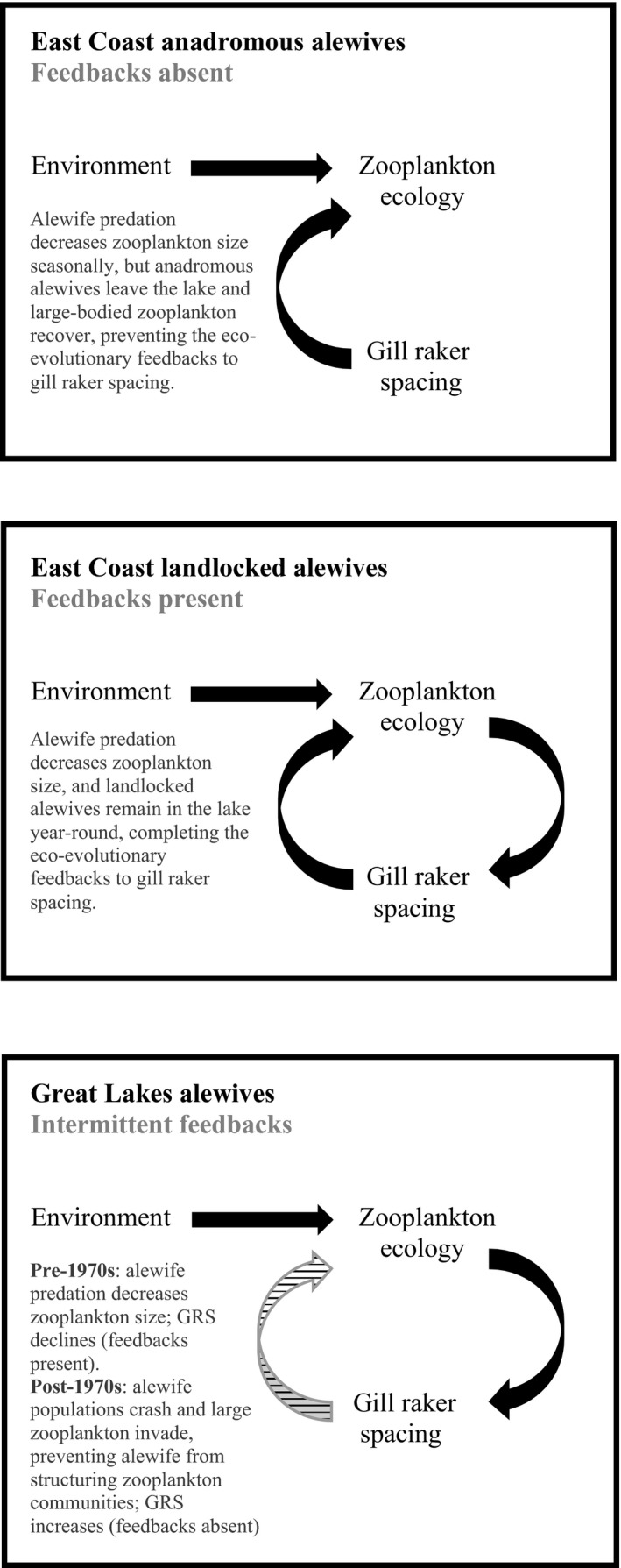
The presence and absence of eco‐evolutionary feedback loops in relation to gill raker spacing (GRS) in respective East Coast anadromous, East Coast landlocked, and Great Lakes alewife populations. The top box displays an absence of consistent feedbacks in the East Coast anadromous population, the middle box displays a presence of consistent feedbacks in East Coast landlocked populations, and the bottom box displays intermittent feedbacks that encapsulate the overall patterns observed in Great Lakes alewives throughout the past 95 years

Our analyses revealed that gill raker spacing in Great Lakes alewives was more similar to the smaller spacing exhibited by East Coast landlocked populations into the 1970s, but that contemporary Great Lakes alewives have gill raker spacing more similar to the East Coast anadromous population. Although phenotypic patterns of gill raker spacing differed between Great Lakes and East Coast landlocked populations, we argue that parallel processes have driven this trait in both sets of landlocked populations. In both systems, alewives shaped the community structure of their prey and subsequently adapted to feed on the shifted prey community (Hutchinson, 1971; Warshaw, 1972; Kohler & Ney, 1981; Palkovacs & Post, 2008; Post et al., 2008; Palkovacs et al., 2014), but a series of fishery management decisions and unintentional introductions of invasive species led to a disruption of the feedback between zooplankton ecology and alewives during and after the 1970s in the Great Lakes alewife populations. Hence, the eco‐evolutionary feedback loops were intermittent throughout the century, with strong feedbacks likely being present from the time of alewife colonization up until the 1970s when alewife populations crashed, and then the absence of feedbacks post‐1970s as a result of low alewife recruitment and the invasion of competitors and large prey items, preventing alewives from structuring zooplankton communities as they once did (Figure 7). More broadly, this finding suggests that the eco‐evolutionary dynamics in which a predator becomes entangled in complex feedback loop with their respective prey may be a common process (e.g., Brunner et al., 2019; Hiltunen et al., 2014; Palkovacs and Post, 2008; Post et al., 2008; Schaffner et al., 2019; Yoshida et al., 2003), yet one that is subject to the same types of contingencies that shape adaptive evolution more generally (Losos et al., 1998; Blount et al., 2018).

Unlike the highly responsive, rapid changes in Great Lakes alewife gill raker spacing, gape width remained relatively stable from initial alewife colonization until the 2010s in Lake Ontario populations, while Lake Michigan alewives showed a consistent increase in this trait from first colonization up until the 2010s (Figure 3). The initial difference in gape width between our first data points for Lake Ontario (1920s) and Lake Michigan (1950s) is approximately 0.4 millimeters, although for both populations contemporary gape width was more similar to East Coast landlocked populations than the larger gape width that occurs in the East Coast anadromous population. One hypothesis to explain why gape width increased in Lake Michigan (ultimately reaching a similar gape width to Lake Ontario) is that over six decades, alewives in Lake Michigan adapted to reduce their gape limitation from consuming mysids (*Mysis relicta*), which range in length from 4 to 20 millimeters (Pothoven, Fahnenstiel, & Vanderploeg, 2010) and provide an energy‐rich prey resource (Gardner, Nalepa, Frez, Cichocki, & Landrum, 1985). Studies in Lake Michigan reveal that alewives have consumed mysids since the 1980s (see review by Bunnell et al., 2015) despite a history of zooplankton size fluctuation and introduced prey species (Pothoven & Vanderploeg, 2004; Stewart et al., 2009). Future research, however, will be needed to explain why even the earliest measurements of gape width in the Great Lakes were markedly lower than what was observed in the anadromous population and remained consistently closer to East Coast landlocked populations.

There are several competing hypotheses that may explain why gape width did not decrease over time or closely correspond to gill raker spacing evolutionary trajectory. We argue the most plausible explanation is that stabilizing selection acted on Lake Ontario alewife gape width and directional selection acted on Lake Michigan alewife gape width over the course of 95 years, resulting in an optimal gape width where an increase or decrease to the gape width may decrease efficiency in prey capture. Using negative pressure created in the buccal cavity, alewives can use a suction motion to selectively pursue prey, typically larger zooplankton, and create a vortex to suction their prey inward (Wainwright et al., 2007). The measured gape width of contemporary Great Lakes alewife populations may represent the optimal vortex to facilitate selective suction feeding. Alternatively, the current gape width may accommodate selective and nonselective feeding mechanisms that shift with alewife size. A study by Janssen (1976) revealed that alewives 114 millimeters TL and less were size‐selective particulate feeders, alewives 124‐152 millimeters were size‐selective and fed by gulping, and alewives larger than 178 millimeters fed by filter‐feeding and were not size‐selective. As feeding modes and prey size selectivity change throughout an alewife’s lifetime, a gape width that can accommodate both large and small prey items may be most advantageous. Another possibility is that the rate of evolution in each trait varies considerably; gill raker spacing may reflect rapid changes, while rates of evolution are much slower in gape width. However, studies in East Coast landlocked populations have demonstrated that significant changes in both gape width and gill raker spacing can occur within 300‐5,000 years (Palkovacs & Post, 2008; Post et al., 2008), suggesting both traits are capable or rapidly evolving. Finally, it is possible that the stasis in gape width in Lake Ontario alewives was a result of reduced genetic variation from a founder effect or population reduction event due to die‐offs. It is also worth noting that the earliest records of alewives in the Great Lakes date to 1873 and our earliest museum specimens used were dated from 1922. It is possible there was an initial shift in gape width that preceded our measurements. Although common garden experiments performed by Palkovacs and Post (2008) demonstrated East Coast anadromous and landlocked alewives maintained differences in gill raker spacing and gape width in the absence of environmental heterogeneity, supporting evidence for a genetic basis of inheritance, phenotypic plasticity in Great Lakes alewives cannot be entirely ruled out.

### 4.2 Body shape evolution

Our results showed the overall body shape of contemporary Great Lakes alewives was more consistent with patterns exhibited by East Coast landlocked populations than in East Coast anadromous alewives. Both Great Lakes populations displayed differing, distinct body morphology; while Lake Ontario fish possessed a more super‐terminal oriented mouth and ventrally emphasized curvature, Lake Michigan fish subsequently displayed a more sub‐terminal oriented mouth and dorsally concentrated curvature. Collectively, Great Lakes alewives had smaller heads, deeper, more robust bodies, and slimmer caudal peduncles than the native anadromous population (Figure 4). While migratory alewife populations require more fusiform, streamlined bodies for efficient hydrodynamics and sustained swimming (Taylor & Foote, 1991), we found that Great Lakes alewives evolved a deeper, less streamlined body shape similar to East Coast landlocked populations. Although changes to Lake Michigan alewives over 62 years (1950s‐2010s) did not show a clear evolutionary pattern and trajectory (Figure 6), Lake Ontario alewives did exhibit a consistent increase in body depth over a period of only 85 years (1930s–2010s; Figure 5). We argue this change in body shape could be due to the cessation of migration and associated reduced energetic demands of long‐distance movement. Our results are consistent with recent studies that found less streamlined bodies associated with a loss or reduction in migration distance (Lahti et al., 2009; Velotta et al., 2018). A recent study by Velotta et al. (2018) showed that body shape changes in independent East Coast landlocked populations of alewives resulted in a reduction in prolonged swimming efficiency that was attributed to the repeated loss of long‐distance migration across populations and that selection for prolonged swimming was expected to be higher in ancestral anadromous alewives than in fish confined to inland lakes. The decrease in prolonged swimming efficiency among East Coast landlocked populations and Great Lakes populations may be related to the energy and resource availability for their respective environments, as marine habitats are typically higher in food availability than freshwater environments (Morgan & Iwama, 1991).

Deeper, more robust bodies may in part be due to the loss of migration, but this change also may be a consequence of inhabiting a novel environment with an assemblage of new predators. Gape limitation is a common defense mechanism (Mihalitsis & Bellwood, 2017) that evolves to prevent a prey fish from fitting into the mouth of a predatory fish, rendering a safe prey‐refuge size that increases with body depth. The introduction of Coho and Chinook salmon into the Great Lakes, along with native predatory fishes (e.g., lake trout), may select for increased body depth. Alternate explanations for a deeper body with a smaller head and more slender caudal peduncle include the possibility that this combination of motility traits makes it functionally easier to capture prey within a new trophic niche, or this novel trait combination is well‐suited for exploiting available resources in the novel environment of the Great Lakes. Our results suggest that while foraging traits (gill raker spacing and gape width) closely track food sources, traits associated with locomotion show parallel evolution among all landlocked populations, despite the profound differences between the Great Lakes ecosystem and the relatively small East Coast lakes.

Our results suggest that alewives have adapted to a novel environment following their colonization of the Great Lakes. After the colonization of the Great Lakes, alewives likely entered an eco‐evolutionary feedback loop remarkably similar to East Coast landlocked populations. A series of major changes in the Great Lakes ecosystem, including the introduction of salmon, dreissenid mussels, and various large‐bodied zooplankton, weakened the feedback loop, reversing the phenotypic trajectory in traits linked to feeding. We propose that this is best described as an intermittent eco‐evolutionary feedback loop. While trophic traits evolved in response to species interactions, body shape in Great Lakes alewife populations remained distinct yet similar to East Coast landlocked populations. Thus, the novel combination of traits found in Great Lakes alewives is a result of a combination of highly parallel trait changes and contingent eco‐evolutionary feedbacks resulting from a complex history of changes in the pelagic ecosystems of the Laurentian Great Lakes.

## Supporting information

Table S1

## Data Availability

Data for this study are available at Dryad: https://doi.org/10.5061/dryad.gb5mkkwmt.
