# Supplementary material for: A century of intermittent eco‐evolutionary feedbacks resulted in novel trait combinations in invasive Great Lakes alewives (Alosa pseudoharengus)
Source: Evol Appl. 2020 Aug 25;13(10):2630–45. doi: 10.1111/eva.13063 (PMC7691454; doi:10.1111/eva.13063)
Supplement: Supplementary file 1 — Table S1 [file EVA-13-2630-s001.docx]

**Supplemental Table S1.** All museum and contemporary Great Lakes specimens and East Coast anadromous and landlocked data used in gill raker spacing, gape width, and geometric morphometric body shape analyses.

| **Life history form** | **Population** | **Museum ID/sampling source** | **Year sampled** | **# of individuals used in GRS/GW analyses** | **# of individuals used in body shape analyses** | **Mean Standard Length (SL) (mm)** |
| --- | --- | --- | --- | --- | --- | --- |
| Great Lakes landlocked | Lake Ontario | ROM-1347 | 1922 | - | 3 | - |
| Great Lakes landlocked | Lake Ontario | ROM-8151 | 1927 | 15 | 15 | 59.64 |
| Great Lakes landlocked | Lake Ontario | UMMZ-89441 | 1929 | 15 | 35 | 49.11 |
| Great Lakes landlocked | Lake Ontario | ROM-007989 | 1932 | 3 | 3 | 78.91 |
| Great Lakes landlocked | Lake Ontario | ROM-12773 | 1940 | 10 | 15 | 41.33 |
| Great Lakes landlocked | Lake Ontario | ROM-19854 | 1948 | 12 | 15 | 43.96 |
| Great Lakes landlocked | Lake Ontario | ROM-19053 | 1948 | 7 | 7 | 59.59 |
| Great Lakes landlocked | Lake Ontario | ROM-18355 | 1948 | - | 3 | - |
| Great Lakes landlocked | Lake Ontario | ROM-27626 | 1970 | 15 | 15 | 112.96 |
| Great Lakes landlocked | Lake Ontario | ROM-44696 | 1984 | 12 | 12 | 123.51 |
| Great Lakes landlocked | Lake Ontario | ROM-94755 | 2013 | 14 | 16 | 124.99 |
| Great Lakes landlocked | Lake Ontario | USGS-446 | 2017 | 59 | 37 | 139.72 |
| Great Lakes landlocked | Lake Michigan | UMMZ-170945 | 1955 | 10 | 16 | 64.83 |
| Great Lakes landlocked | Lake Michigan | INHS-648 | 1960 | 5 | 15 | 132.75 |
| Great Lakes landlocked | Lake Michigan | INHS-4232 | 1963 | 5 | 13 | 127.1 |
| Great Lakes landlocked | Lake Michigan | INHS-611 | 1964 | - | 8 | - |
| Great Lakes landlocked | Lake Michigan | INHS-4250 | 1964 | 10 | 24 | 57.99 |
| Great Lakes landlocked | Lake Michigan | INHS-4283 | 1964 | 5 | 14 | 67.39 |
| Great Lakes landlocked | Lake Michigan | INHS-4268 | 1964 | 10 | 22 | 81.87 |
| Great Lakes landlocked | Lake Michigan | INHS-4311 | 1967 | - | 6 | - |
| Great Lakes landlocked | Lake Michigan | FMNH-75800 | 1969 | 11 | 11 | 105.32 |
| Great Lakes landlocked | Lake Michigan | FMNH-85046 | 1977 | 10 | 11 | 145.96 |
| Great Lakes landlocked | Lake Michigan | FMNH-86323 | 1977 | 15 | 15 | 128.75 |
| Great Lakes landlocked | Lake Michigan | FMNH-85053 | 1977 | 5 | 8 | 151.02 |
| Great Lakes landlocked | Lake Michigan | FMNH-97955 | 1982 | 9 | 10 | 38.45 |
| Great Lakes landlocked | Lake Michigan | INHS-56897 | 1979 | - | 4 | - |
| Great Lakes landlocked | Lake Michigan | INHS-67690 | 1982 | - | 3 | - |
| Great Lakes landlocked | Lake Michigan | INHS-88702 | 1984 | 5 | 8 | 65.27 |
| Great Lakes landlocked | Lake Michigan | INHS-57074 | 2000 | 5 | 9 | 132.72 |
| Great Lakes landlocked | Lake Michigan | DDB_2017 | 2017 | 14 | 13 | 115.42 |
| East Coast landlocked | Crystal Lake | Palkovacs and Post 2008 | 2004, 2005 | 26 | - | - |
| East Coast landlocked | Amos Lake | Palkovacs and Post 2008 | 2004, 2005 | 20 | - | - |
| East Coast landlocked | Uncas Pond | Palkovacs and Post 2008 | 2004, 2005 | 22 | - | - |
| East Coast landlocked | Saltonstall Lake | Palkovacs and Post 2008 | 2004, 2005 | 25 | - | - |
| East Coast landlocked | Long Pond | Palkovacs and Post 2008 | 2004, 2005 | 16 | - | - |
| East Coast landlocked | Mashapaug Lake | Palkovacs and Post 2008 | 2004, 2005 | 26 | - | - |
| East Coast landlocked | Pattagansett Lake | Palkovacs and Post 2008 | 2004, 2005 | 76 | - |  |
| East Coast landlocked | Pattagansett Lake | Jones *et al.* 2013 | 2009 | - | 44 | - |
| East Coast landlocked | Quonnipaug Lake | Palkovacs and Post 2008 | 2004, 2005 | 90 | - | - |
| East Coast landlocked | Quonnipaug Lake | Jones *et al.* 2013 | 2009 | - | 23 |  |
| East Coast landlocked | Rogers Lake | Palkovacs and Post 2008 | 2004, 2005 | 80 | - |  |
| East Coast landlocked | Rogers Lake | Jones *et al.* 2013 | 2009 | - | 28 | - |
| East Coast landlocked | Bride Lake | Palkovacs and Post 2008 | 2004, 2005 | 56 | - |  |
| East Coast anadromous | Bride Lake | Jones *et al.* 2013 | 2009 | - | 62 | - |
| East Coast landlocked | Dodge Pond | Palkovacs and Post 2008 | 2004, 2005 | 49 | - |  |
| East Coast anadromous | Dodge Pond | Jones *et al.* 2013 | 2009 | - | 79 | - |
| East Coast anadromous | Gorton Pond | Palkovacs and Post 2008 | 2004, 2005 | 59 | - | - |
| East Coast anadromous | Upper Mill Pond | Jones *et al.* 2013 | 2009 | - | 40 | - |
